# Gender differences in brain areas involved in silent counting by means of fMRI 

**DOI:** 10.1186/1753-4631-4-S1-S2

**Published:** 2010-06-03

**Authors:** Olivera B Šveljo, Katarina M Koprivšek, Miloš A Lučić, Mladen B Prvulović, Milka Ćulić

**Affiliations:** 1 Diagnostic Imaging Centre, Institute of Oncology, Instituski put 4, Sremska Kamenica, Serbia; 2 Institute for Biological Research “Siniša Stanković”, University of Belgrade, Bulevar despota Stefana 142, Belgrade, Serbia

## Abstract

**Background:**

Pattern of brain asymmetries varies with handedness, gender, age, and with variety of genetic and social factors. Large-scale neuroimaging analyses can optimize the detection of asymmetric features and confirm the factors that might modulate pattern of brain asymmetries. We attempted to evaluate eventual differences between genders in hemodynamic responses to a simple language task.

**Methods:**

12 healthy right-handed volunteers (age 24-46), 6 men and 6 women underwent fMRI scanning while performing the simple cognitive - language processing task – silent number counting in Serbian.

**Results:**

Group analysis of hemodynamic responses shows activation in expected brain language areas of inferior frontal gyrus (IFG) and superior temporal gyrus (STG) in both hemispheres. In the male group, aside from dedicated language areas in IFG and STG, activation was noted in right frontal region and interhemispheric supplementary motor area. On the other hand, in the female group, besides activation in dedicated language areas, activation was noted, in right hippocampus, limbic brain and cerebellum bilaterally.

**Conclusions:**

Our results on differences in silent counting by means of fMRI suggest that those differences may be based on different brain pattern activation in men and women. The relation between performance, strategies and regional brain activation should be the topic of further studies when considering not only gender differences in language processing but also differences that may be attributed to the variations in the task details, stimuli, and the stimulus presentation methods.

## Background

Sexual dimorphism in humans and its implications to gender behaviour has been in focus of philosophers and researchers for centuries. Through the whole 20^th^ century it has been generally accepted that there are no significant differences in brain anatomy between genders but females are considered better in performing some tasks and male in some other. In general, females are considered better in languages performances than males and males are considered better in visual-spatial tasks [1]. 

Considering the classical language regions (Broca's area and Wernicke's area) as recently  reviewed [2], fMRI studies report sex/gender related differences in language production [3-5] as well as in language perception [6-8]. However, on the basis of fMRI imaging data, the number of brain areas described as being involved in language processing has increased; activation in the context of the sex/gender variable has been found in the angular gyrus, in prefrontal, thalamocapsular, retrosplenial, and cerebellar regions [6], and in the (pre-) cuneus and cingulate areas [9]. In addition, bi/lateralisation effects due to sex/gender in other than classical language areas were shown in fusiform regions [10,11].

Since the publishing of one of the most cited studies [12], it is accepted that females’ language network considers the inferior frontal regions of both hemispheres, while males’ language function is strongly lateralised to the left inferior frontal area, and that these variations exist at the level of phonological processing. There is also an evidence [13] of hemispheric lateralisation for echoic memory trace with long lifetimes which depend on gender and handedness while the underlying physiological basis for speech and language processing remains a major challenge for cognitive neuroscience. Our aim was to investigate possible differences in the language processing in adult healthy volunteers for silent counting as a simple word generation task by means of fMRI and preliminary account on this study has already appeared [14].

## Methods

### Participants

Twelve right-handed healthy native Serbian-speaking volunteers - 6 men and 6 women, between 24 and 46 years of age (mean age=35.17±9.28 for men and 34.83±6.27 for women), underwent fMRI examination. All participants gave informed consent to undergo scanning on 3T Siemens Trio MR unit. The study has been approved by the Ethical Committee of the Institute of Oncology of Vojvodina at Sremska Kamenica. 

### Experimental protocol and data acquisition

The simple language task was just silent counting forward from 1 to 30, in Serbian, and the control state was the complete rest. Subjects were instructed and rehearsed in the task before scanning, asked to be quiet in the scanner having headphones and thereafter positioned in the gantry. 

Both anatomical and functional MR images were obtained for each subject. The functional images were obtained in axial planes at 3 seconds interval while subject alternatively rested and performed specified language task for 30 seconds. Technical parameters for the images included: TR 3000, TE 30, matrix 64x64, field of view 240, and slice thickness 3 mm. To avoid activations of areas that are not necessary in word generation process as well as head movements, just a simple self paced silent word generation task has been used. During the active state, subjects were asked to generate silently about one number per second, in consecutive order starting with number 1. Start and stop instructions were given through headphones. After scanning, subjects were asked whether or not they performed tasks successfully. 

### Image processing

For generation of fMRI activation map for each subject as well as for group analysis, the software FSL, FMRIB from Oxford, UK (**S**oftware **L**ibrary, **F**unctional **MRI** of the **B**rain) has been used [15]. For generation of fMRI activation map for single subject analysis FEAT (FMRI Expert Analysis Tool), part of the FSL, has been used. Standard steps in pre-processing were applied: motion correction was done by the MCFLIRT (Motion Correction using FMRIB`s Linear Image Registration Tool), non-brain removal by the BET (Brain Extraction Tool) [16], spatial smoothing by a Gaussian kernel of FWHM 5mm. Mean-based intensity normalisation of all volumes was done by the same factor, high-pass temporal filtering (Gaussian-weighted last-squares straight line fitting, with sigma = 50.0 s). Time-series statistical analysis was carried out using the FILM (FMRIB`s Improved Linear Model; [17]. The statistics images were initially corrected for multiple comparisons using cluster threshold determined by Z-scores >2.3, and a corrected cluster significance of P=0.05. Registration to standard images (MNI 125) was carried out using the FLIRT (FMRIB`s Linear Image Registration Tool) [18,19]. 

Higher-level analysis was carried out using FLAME (FMRIB's Local Analysis of Mixed Effects) stage 1 only [20,21]. Z (Gaussianised T/F) statistic images were using clusters determined by Z>1.7 and at the corrected cluster significance threshold of P=0.05 [22]. 

## Results

**Figure 1:**
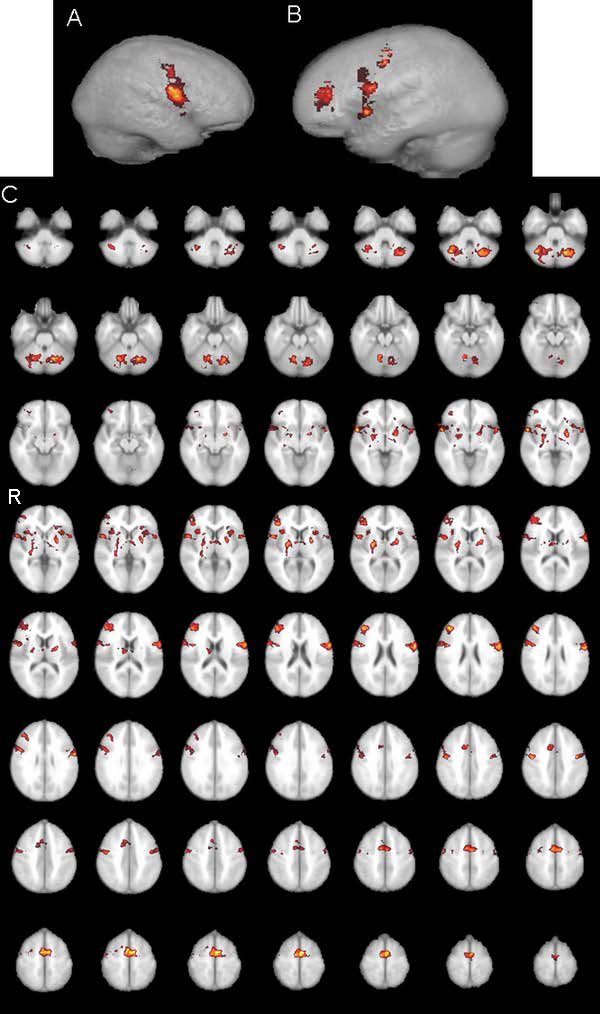
**fMRI whole group analysis** Statistical fMRI map activation patterns in the whole group: A) in the left hemisphere; B) in the right hemisphere; C) in transversal projections through the whole brain. (cluster threshold activation images (min. red, max yellow) Z statistic range automatically calculated by default 1.7< Z< 4.3))

**Figure 2:**
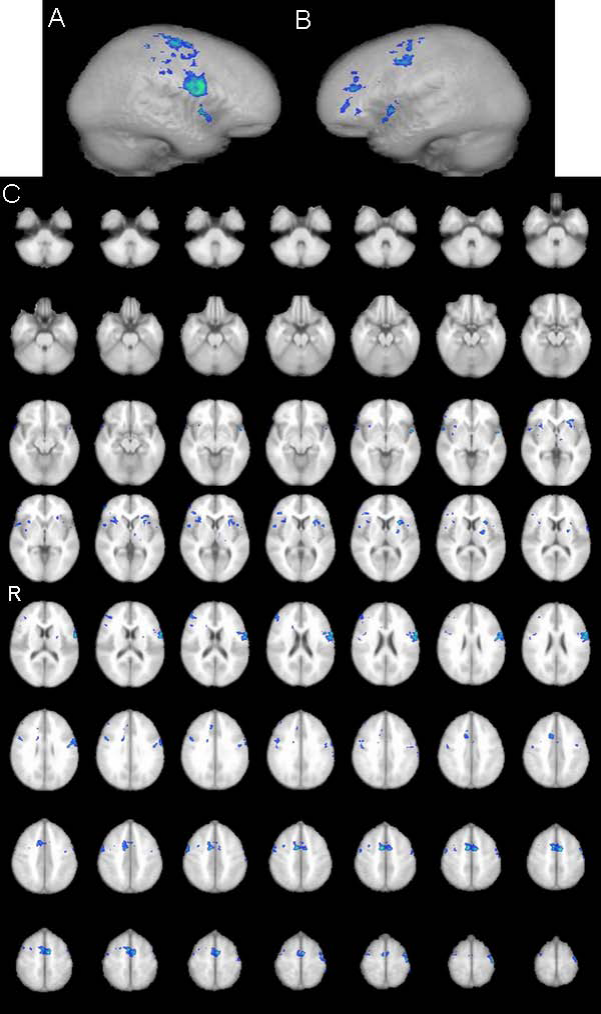
**fMRI male group analysis** fMRI map activation patterns in the group of male subjects: A) in the left hemisphere; B) in the right hemisphere; C) in transversal projections through the whole brain (cluster threshold activation images (min. blue, max green) Z statistic range automatically calculated by default (1.7< Z< 3.4))

**Figure 3:**
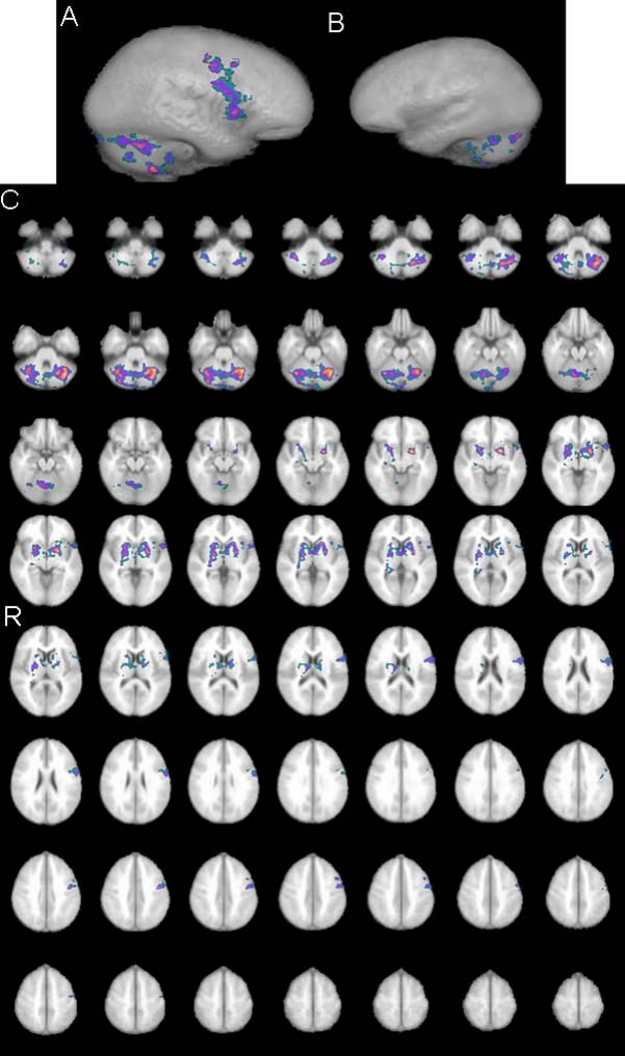
**fMRI female group analysis** fMRI map activation patterns in the group of female subjects: A) in the left hemisphere; B) in the right hemisphere; C) in transversal projections through the whole brain. (cluster threshold activation images (min. green, max yellow) Z statistic range automatically calculated by default 1.2< Z< 2.9)

**Figure 4:**
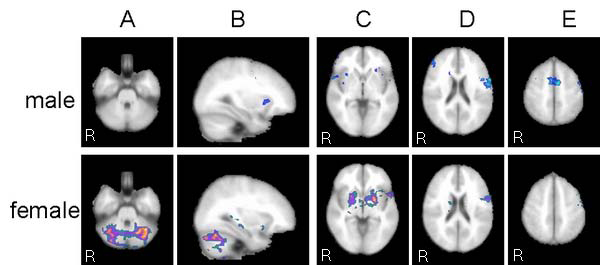
**fMRI gender differences** Major differences between female and male subjects were found in A) cerebellum, B) right hippocampus, C) limbic region, D) right frontal area, and E) supplementary motor area.

Group analysis for activations by means of fMRI across all subjects is shown on Figure 1; Table 1 provides an overview of all significantly activated regions. Considering all 12 subjects significant activations were found in dedicated language areas of inferior frontal gyrus (IFG) and anterior part of superior temporal gyrus (STG) bilaterally, as well as activations in right frontal region, right hippocampus, limbic brain and cerebellum bilaterally. For male group higher-level analysis was carried out using same pre-processing steps as for the whole group higher-level analysis (FLAME stage 1 only, Z (Gaussianised T/F) statistic images were at the threshold using clusters determined by Z>1.7 and corrected cluster significance threshold of P=0.05. Active regions, when considering only male group, were found in IFG and STG bilaterally, as well as in right frontal region, interhemispheric supplementary motor area, and left limbic brain (Figure 2, Table 1). Higher-level analysis for the female group was carried out using same pre-processing steps except that statistic images were at the threshold using cluster determined by Z>1.2 (with Z >1.7 - activation only in cerebellum could be seen) while corrected cluster significance threshold remained the same P=0.05. In female group, strong left language lateralisation has been found i.e. activation of language areas were found only in left hemisphere (Figure 3A). Besides activation in dedicated language areas in female group, active region was noted in right hippocampus, limbic brain and cerebellum bilaterally (Figure 3, Table 1). Notable differences in fMRI brain activation patterns for a simple language task (counting) between male and female subjects were found in cerebellum, right hippocampus, right frontal region and supplementary motor area (Figure 4). Higher-level analysis for direct comparison between male and female group (i.e. male > female, Z>1.7, P=0.05) confirmed differences in brain pattern activation between male and female subjects while differences in activations of right hippocampus, right limbic region and cerebellum between female and male group (i.e. female > male Z>1.2, P=0.05) could not be confirmed, probably because of generally better hemodynamic responses in male than in female group. 

**Table 1:** MNI coordinates of activated regions

		MNI coordinates			
**Region**	**L/R**	***x***	***y***	***z***	**Z-score**

SMA - all		-2	-4	60	4.25
M		-2	-4	60	2.92
F		-	-	-	-

Precentral gyrus	R-all	44	-8	42	2.56
	M	50	-8	38	2.54
	F	-	-	-	-
	
	L-all	-46	-8	42	2.56
	M	-50	-8	36	2.12
	F	-50	-4	42	1.77

Prefrontal cortex	R-all	42	36	22	3.72
	M	48	36	20	2.10
	F	-	-	-	-

Limbic brain	R-all	24	-8	10	3.16
	M	-	-	-	-
	F	24	6	-8	2.16
	
	L-all	-20	-6	10	3.16
	M	-24	-6	8	2.54
	F	-24	-2	2	2.57

Inferior frontal gyrus	R-all	60	4	20	2.54
	M	54	-2	24	2.11
	F	-	-	-	-
	
	L-all	-60	2	20	3.71
	M	-60	2	20	2.53
	F	-58	2	20	2.17

Hippocampus	R-all	32	-24	-8	2.55
	M	-	-	-	-
	F	30	-24	-10	1.76

Superior temporal gyrus	R-all	58	8	-6	3.68
	M	58	10	-4	2.92
	F	-	-	-	-
	
	L-all	-54	4	-4	2.51
	M	-52	4	-4	2.93
	F	-54	10	-6	2.14

Cerebellum	R-all	36	-64	-30	3.70
	M	-	-	-	-
	F	36	-64	-30	2.55
	
	L-all	-20	-68	-30	3.70
	M	-	-	-	-
	F	-20	-68	-30	2.16

MNI coordinates of activated regions for all subjects and female’s and male’s group (significant at P=0.05)

## Discussion

The pioneer work of Broca and Wernicke revealed the primary language areas located in inferior frontal and temporal part of the cerebral cortex in the dominant hemisphere, and accordingly, the classical model of language generation has been defined. The results on translation and language switching by the use of positron emission tomography [23] revealed contrasting patterns of activation for translation and switching. Translation, but not switching, increased activity in the anterior cingulate and subcortical structures whilst decreasing activation in several other temporal and parietal language areas associated with the meaning of words. Translation also increased activation in regions associated with articulation (the anterior insula, cerebellum and supplementary motor area). In contrast, switching the sensory input language processing resulted in activation of Broca's area and the supramarginal gyri, areas associated with phonological recoding. fMRI studies reveal many aspects of language generation network, too. Imaging research studies showed that brain areas which participate in language brain network depend on type of task presentation (visually, auditory), and task itself (language perception, language production, semantic, phonologic, orthographic aspects) [11,24,25]. 

In this study we used silent fluent word generation task without visual or auditory stimuli. Our intention was to avoid any unnecessary activation of language generation network as well as to minimise cognitive and linguistic components underlying each task. We expected certain differences between genders and considering primary language areas, differences between genders were in the range of other fMRI language research studies [2]. We found strong left language lateralisation in female subjects and bilateral representation of Broca’s area in male subjects. Distinctive differences in activations of supplementary motor area, prefrontal region, and certain cerebellar and limbic regions that we have found were completely unexpected. We would point out the cerebellar involvement, from anatomic and functional points of view. It is now recognized that the cerebellum not only controls motor coordination but also represents an essential component of the brain mechanisms in cognition based on predictive and preparative functions of the cerebellum caused by connections between the cerebellum and the cerebral cortex [26-28]. Brain activations observed during simple speech tasks indicate the superior paravermal cerebellum as more active for consonant-vowel syllables compared with vowels, perhaps due to increased timing constraints for consonant production [29]. It was also suggested [4] that female advantage in certain executive speech tasks, such as verbal fluency could be attributed to different processing strategies for lexical verbal fluency. Peculiarities of our study related to the others on language processing were verbalisation of symbolic meaning (numbers) and task representations. Actually, we used self paced paradigm for “over learned” task of counting that included basic memory/learning aspects, and differences that we found could be due to the different strategies between genders in simple memory retrieval/learning task. Axmacher et al and Özetkin et al. showed in there recent studies the interference of working and long term memory in memory retrieval and memory formation [30,31] process. Axmacher et al also showed different brain pattern activation for tasks with high working memory load and tasks with low memory load as well as different brain pattern activation in the case of successful memory retrieval and memory failure. Prefrontal cortex and hippocampal structures are usually related to memory processing and cognitive strategies. It has been shown that prefrontal cortex is involved in monitoring and manipulation of information in working memory [32]. On the other hand, medial temporal lobe structures are usually related to long term memory functions [30,33]. Our results may suggest that gender related differences in language processing reflect specific cognitive and executive strategies as a response to certain stimuli and that this should be considered in future studies. However, meta-analysis [34] implies that the putative sex difference in language lateralization may be absent at the population level, or may be observed only with some, as yet not defined, language tasks. It is interesting that Wang at al. in their study of gender differences to psychological stress [35] have found similar differences in activation of prefrontal areas and limbic regions between males and females but with the more demanding task. We have to point out that the chosen simple task – silent counting in our study was certainly not stressful because it was suggested that for some tasks, stress evokes sex differences and that these differences are mediated largely by interactions between stress and sex hormones [36]. 

## Conclusions

Although through the whole last century the strong believe that the behavioural differences between genders are mainly based on different biosocial conditions has been present, evolutional psychology at the beginning of current century proposed a new hypothesis that the sexual dimorphisms in humans has developed from different gender related strategies in the realisation of the same tasks through the evolution [37]. In our opinion differences in brain pattern activation between female and male subjects that we found, could support this hypothesis. Of course, one should be cautious when interpreting studies that purport to have identified regions of difference between groups, whether those groups are divided by sex or by any other criterion [38].

## Competing interests

The authors declare that they have no competing interests.

## Authors' contributions

All authors contributed equally to this work.
